# From ERα66 to ERα36: a generic method for validating a prognosis marker of breast tumor progression

**DOI:** 10.1186/s12918-015-0178-7

**Published:** 2015-06-17

**Authors:** Clémence Chamard-Jovenin, Alain C. Jung, Amand Chesnel, Joseph Abecassis, Stéphane Flament, Sonia Ledrappier, Christine Macabre, Taha Boukhobza, Hélène Dumond

**Affiliations:** CNRS-Université de Lorraine, UMR 7039, Centre de Recherche en Automatique de Nancy, BP70239, F-54506 Vandœuvre-lès-Nancy, France; EA 3430, Centre Paul Strauss, 3 rue Porte de l’Hôpital, 67000 Strasbourg, France

**Keywords:** ERalpha36, Breast tumor, Retrospective study, Gene network identification, Metastatic potential, Nonlinear correlation, Distance based tumor classification

## Abstract

**Background:**

Estrogen receptor alpha36 (ERalpha36), a variant of estrogen receptor alpha (ER) is expressed in about half of breast tumors, independently of the [ER+]/[ER-] status. *In vitro*, ERalpha36 triggers mitogenic non-genomic signaling and migration ability in response to 17beta-estradiol and tamoxifen*. In vivo*, highly ERalpha36 expressing tumors are of poor outcome especially as [ER+] tumors are submitted to tamoxifen treatment which, in turn, enhances ERalpha36 expression.

**Results:**

Our study aimed to validate ERalpha36 expression as a reliable prognostic factor for cancer progression from an estrogen dependent proliferative tumor toward an estrogen dispensable metastatic disease. In a retrospective study, we tried to decipher underlying mechanisms of cancer progression by using an original modeling of the relationships between ERalpha36, other estrogen and growth factor receptors and metastatic marker expression. Nonlinear correlation analyses and mutual information computations led to characterize a complex network connecting ERalpha36 to either non-genomic estrogen signaling or to metastatic process.

**Conclusions:**

This study identifies ERalpha36 expression level as a relevant classifier which should be taken into account for breast tumors clinical characterization and [ER+] tumor treatment orientation, using a generic approach for the rapid, cheap and relevant evaluation of any candidate gene expression as a predictor of a complex biological process.

**Electronic supplementary material:**

The online version of this article (doi:10.1186/s12918-015-0178-7) contains supplementary material, which is available to authorized users.

## Background

Worldwide, breast cancer remains one of the main causes of cancer-induced morbidity and mortality in women. Breast tumors are usually classified according to clinical parameters (size, grade, lymph node extension) and molecular expression status (ER, PR, HER2, Claudin) [1]. Such a classification allows clinicians ordering the appropriate treatment. For instance, ER-positive/negative ([ER+]/[ER-]) status refers to the expression of the 66kDa nuclear estrogen receptor α (ERα66) in tumors, which are consequently cured by endocrine therapeutic agents such as tamoxifen. Nevertheless, about 30 % therapeutic failure is observed due to unclear resistance mechanisms [2].

Until the recent identification of new membrane bound estrogen receptors, ERα66 has been considered as the sole functional estrogen receptor in hormone sensitive breast tumor. In 2005, Wang and colleagues [3] cloned a 36-kDa variant of ER-alpha (ERα36) which lacks both AF-1 and AF-2 transcription activation domains but retains a truncated ligand-binding domain, suggesting that ERα36 may have a spectrum of ligand selectivity different from ERα66.

ERα36 is generated from a promoter located in the first intron of the ESR1 gene, indicating that ERα36 expression is regulated independently from ERα66. This is consistent with the finding that ERα36 protein is present in about 40 % of [ER+] and [ER-] breast tumors.

ERα36 triggers membrane-initiated mitogenic estrogen signaling through non-genomic pathways not only in breast, but also in gastric and laryngeal cancer cells both *in vitro* and *in vivo* [4–7]*.* In the [ER+] MCF-7 breast tumor cell line, ERα36 overexpression leads to tamoxifen resistance and enhances metastatic potential [8, 9]. Thus, tamoxifen does not act as a drug for cancer treatment but serves as an ERα36 agonist, triggering proliferation, migration and invasion. The adverse effect of tamoxifen in ERα36 highly expressing [ER+] breast tumors may explain why the affected patients display poor outcome and require chemotherapy but not endocrine therapy [10].

These findings raise the possibility that, *in vivo*, enhanced ERα36 expression could drive the growth status switch from estrogen dependent mitogenic signaling to estrogen dispensable migration/invasion ability and consequently stimulates cancer progression. Therefore, we designed a generic method to validate the hypothesis that ERα36 expression may serve as a reliable therapeutic response prognosis marker for breast cancer patients.

A retrospective study was performed on 118 breast tumor samples in which the expression of genes involved in non-genomic estrogen response as well as metastatic process was analyzed. Potential relationship between these genes was modeled by using nonlinear correlation analyses, mutual information associated to significance analysis [11, 12], which are proven to be more accurate than linear statics techniques even if the latter are simpler to implement [13–17]. These models are represented by so-called “gene co-regulation graphs” which can be drawn for any consistent subclass of the considered 118 samples. Then, we used a metric comparing two gene co-regulation graphs to search the optimal value of ERα36 expression providing two distinct populations from a gene network point of view. The two obtained graphs were compared and the differences appeared to be of biological significance.

## Results

### [ER+] versus [ER-] gene networks

**Fig. 1:**
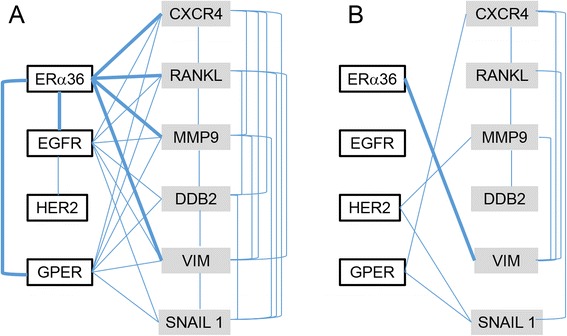
Gene expression network modeling in [ER+] and [ER-] samples. Graphs were designed by computing nonlinear correlation and mutual information between each gene expression pair in either ER-positive (**a**) or ER-negative (**b**) samples. The vertices represent genes. The edges linking the vertices indicate that independence between gene expressions is less than 0.05 and links for ERα36 are in bold. *P*-values are given in Additional file 1: Table S1A and Additional file 2: Table S1B, respectively

Since breast tumors are usually classified according to their hormone receptor status, tumor samples were first split into two classes according to their respective [ER] status, thus defining a first group of 60 ERα66 expressing samples ([ER+]), and a second group of 58 samples devoid of ERα66 expression ([ER-]). [ER+] breast cancer cell lines such as MCF-7 are considered non metastatic and weakly express ERα36 whereas [ER-] cell lines such as MDA-MB-231 or MDA-MB-235 are highly metastatic and display higher levels of ERα36 expression. In order to assess if such a link between ERα36 expression level and metastatic ability may be observed *in vivo*, nuclear (ERα66) or membrane-associated estrogen receptors (ERα36, GPER), their counterparts in non-genomic estrogen signaling (EGFR, HER2) as well as metastatic marker (SNAIL1, CXCR4, RANKL, VIM and MMP9) mRNA expression levels were determined by real-time PCR analyses. Among the growing amount of biomarkers related to the ER status (DDB2), the migration/invasion process (MMP9, VIM, CXCR4, RANKL, SNAIL) or the estrogen-response pathways (GPR30, EGFR), those listed above were picked up because they were previously shown to be related to ERα36 [18–20]. Then, we identified the gene networks for each class of tumors by using nonlinear correlation analyses and transfer entropy computation (see Additional file 1: Table S1A and Additional file 2: Table S1B). The processed data obtained from [ER+] samples indicated that ERα36 was a key node of a complex gene network, which involves other steroid and growth factor receptors as well as metastatic markers as a whole (Fig. 1a). On the other hand, ERα36 was connected to the single metastatic marker VIM in the [ER-] network (Fig. 1b). These huge differences displayed by the two networks implied different functioning modes according to the tumor [ER] status and suggested that there could be a quantifiable link between ERα36 position into the network and/or its expression level and tumor metastatic progression.

### ERα36 based classification of breast tumor samples

To check if ERα36 mRNA expression level could be a relevant classifier of a particular breast tumor phenotype, we drew a gene network for each ERα36 expression value. Then, we quantified the differences between the networks as a function of ERα36 relative expression, and designed a metric playing the role of a distance between graphs. The metric is an integer number standing for the structural differences between two graphs. More precisely, we compared the edges in the two graphs: when an edge existed in a graph and not in the other the distance was incremented with 1, if the edge existed in both graphs but did not represent the same linking way, the distance was incremented with 2. The obtained distance is then a metric.

**Fig. 2:**
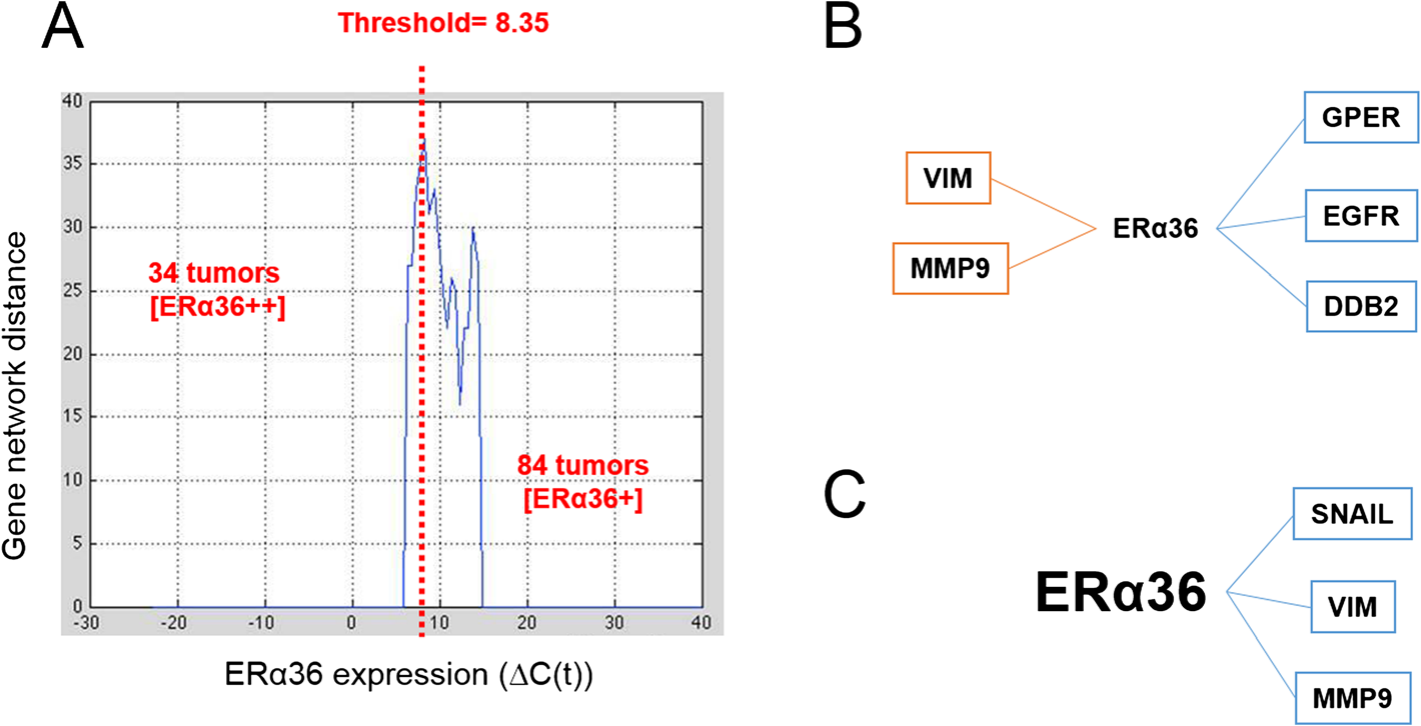
Gene expression network modeling depending on ERα36 expression level. **a** Network distance characterization as a function of ERα36 expression level (see text for details). Expression level varied between 0 and 20 in the samples expressing ERα36 (x-axis). With step of 0.5 on ERα36 expression level, population was divided into two sub-groups for which networks were computed. A distance between corresponding networks was calculated (y-axis). The ERα36 expression threshold corresponding to the most different gene networks was computed and was equal to 8.35. **b**–**c** Graphs were designed by computing nonlinear correlation and mutual information between each gene expression pair in either low ERα36 [ER α36+] expressing (**b**) or high ERα36 [ERα36++] expressing (**c**) samples. The vertices represent genes. The edges linking the vertices indicate that independence between gene expressions is less than 0.05. Positive correlations are in blue and negative correlations in red. Correlation values are given in Additional file 3: Table S2A and Additional file 4: Table S2B and *P*-values in Additional file 5: Table S3A and Additional file 6: Table S3B, respectively

According to this metric, we determined the best threshold for ERα36 to subdivide the samples into two populations, in order to obtain the most different networks probably defining the most different tumor phenotypes related to ERα36 expression (Fig. 2a). Among ERα36 expressing samples, the “best” threshold (which leads to the highest network difference score) was ΔC (t) = 8.35 and allowed to segregate a high ERα36 expressing class ([ERα36++]) of 24 tumors and a low ERα36 expressing class ([ERα36+]) of 84 tumors.

### ERα36 and metastatic progression

In a last step, the previous modeling procedure was applied to either [ERα36+] or [ERα36++] subgroups. When ERα36 expression was low (Fig. 2b), it was clearly related to other receptors (GPER, EGFR) and DDB2 as well as inversely correlated to metastatic markers (MMP9, VIM) (see Additional file 3: Table S2A and Additional file 4: Table S2B). Conversely, in the context of a high ERα36 expression (Fig. 2c), the network indicated a positive relationship to metastatic markers (SNAIL1, VIM and MMP9) independent from other receptors (see Additional file 5: Table S3A and Additional file 6: Table S3B).

## Discussion

In the present study, we examined ERα36 expression in breast tumor specimens from 118 patients. We report that the majority of [ER+] tumors also express high levels of ERα36.

In a previous clinical study, ERα36 expression was shown to correlate with poor outcome in patients with [ER+] tumors treated by tamoxifen and the same tendency was observed in patients with [ER-] tumors [10]. Therefore, a high level of ERα36 expression seemed to be an unfavorable factor of survival in breast cancer patients, independently of ER status. Besides, recent *in vitro* data indicate that ERα36 expression (i) controls metastatic potential in [ER-] HCC38 cells and (ii) confers estrogen-hypersensitivity to [ER+] MCF-7 cells [9, 18]. In order to confirm that ERα36 can trigger the progression of breast cancer in the primary tumor as well as during metastasis and to characterize the underlying mechanisms of high ERα36-dependent phenotypes, we developed modeling tools. Expression analyses and network modeling of estrogen and growth factor receptor encoding genes, well known markers involved in tumor cell migration or invasion, and selected ERα36 target genes [18] suggest that ERα36 could be a key node of estrogen responsive pro-metastatic gene network in [ER+] tumors. These results are in line with recent *in vitro* analyses in MCF-7 cells, which show that the activation of ERα36 expression triggers adaptive changes characterized by enhanced survival and migration during acquired tamoxifen resistance process [8, 21]. Similar data were obtained from endometrial cancer cells wherein ERα36 was shown to promote tamoxifen agonist action *via* the MAPK/ERK and PI3K/Akt pathways [22–24]. Taken together, our results and others clearly suggest that [ER+] tumors highly expressing ERα36 should not be cured by tamoxifen because the treatment could drive metastatic progression.

The developed approach to validate ERα36 as relevant prognostic marker is quite generic and can be applied to other genes as well as to a subset of genes G_0_. Indeed, the only modification, in this case, is to consider that we search for the maxima of multivariable function. Then, a classification can be done according to the expression of each gene to obtain 2^n^ classes, where n is the cardinality of the considered subset G_0_. Moreover, the robustness of the proposed method is attested by the fact that we proceed as described in [25], by using a shuffling method which generates more than 20 000 data for each of the dependency computation done between each pair of the studied genes.

Among the genes tested in this study, ERα36 was identified as the best classifier candidate based on its ability to discriminate between two separate networks: one connecting ERα36 to membrane receptors and the second relating ERα36 expression to those of metastatic markers. Therefore, comprehensive analysis and modeling of gene expression combined to colocalization analysis of ERα36 and ERα66 in breast tumors will contribute to characterize the cascade and timing of events that trigger ERα36 expression during [ER+] metastatic tumor progression.

## Conclusions

In conclusion, this study (i) identifies ERα36 as a relevant classifier whose expression level should be taken into account for breast tumors clinical characterization and [ER+] tumor treatment orientation, (ii) confirms *ex vivo* previous *in vitro* data connecting high ERα36 expression to enhanced expression of migration/invasion markers and (iii) generates a novel approach for the rapid, cheap and relevant evaluation of any candidate gene expression as a predictor of a complex biological process.

## Methods

### Patients

Tumor specimen from 118 women with primary breast cancer expressing the canonical long form of ERα (ERα66) [ER+] or not [ER-] were collected between 1980 and 1998, stored in the Paul Strauss Cancer Center bio-bank and used with the patients’ verbal informed consent with the approval of the hospital ethic committee. Since the tumor pieces used in this study were regarded as post-operative waste materials, verbal consent was recorded by the surgeon during the preoperative examination. The Hospital Ethic Committee for Clinical Research localized into the Paul Strauss Center for Anticancer Research, 3 rue Porte de l’Hôpital, 67000 Strasbourg, France, approved the procedure. 60 [ER+] as well as 58 [ER-] tumor samples were included in the retrospective study. Immediately after resection, one half of each tumor was cryogenized into liquid nitrogen whereas the other part was fixed in 4 % formalin and further used for immunohistological analyses. [ER] status was assayed by standard ligand binding assay. In short, snap frozen tumor samples were pulverized and cytosols were extracted by ultracentrifugation. Human serum albumin was used as a standard control for protein normalization. Cytosol (10 μL) was incubated with 5 nmol/L [H^3^] estradiol. After incubation, 100 μL supernatant were transferred to an isoelectric focusing gel, in order to separate bound, unbound and unspecifically bound hormone. Samples with >10 fmol/mg bound ER were considered to be [ER+].

### RT-QPCR analysis

ERα66, ERα36, GPER, EGFR and HER2, as well as SNAIL1, CXCR4, RANKL, DDB2, VIM and MMP9 expression levels were determined by real-time PCR analyses. Large ribosomal protein (RPLPO) encoding gene was used as a control to obtain normalized values. Primers are listed in [see Additional file 7: Table S4]. Assays were performed at least in triplicate, and the mean values were used to calculate expression levels, using the ΔC (t) method referring to RPLPO housekeeping gene expression. Briefly, total RNA was extracted using RNeasy Plus Universal tissue Mini (Qiagen, Courtabœuf, France) and reverse transcribed (GoScript Reverse Transcription System, Promega, Charbonnières-les-Bains, France). Real-time PCR analyses were then performed by using iTaq Universal SYBR Green Supermix (Bio-Rad, France) in Opticon2 thermocycler (Bio-Rad) as described elsewhere [26].

### Statistical analysis and modeling

Mathematical modeling of biological processes has recently emerged and developed as an essential tool to help cancer biologists and clinician pathologists improving personalized diagnosis, therapy and prognosis. Mainly, the first step in many gene regulation network-modeling task is the identification of the co-regulated or co-expressed genes. To this purpose, most of the works are based on a linear correlation computation and statistical hypothesis tests. Nevertheless, these tools do not detect nonlinear relationship between gene expressions, which is generally the case [13, 14]. That is why we propose to use nonlinear correlation and conditional mutual information techniques on the gene expressions in order to detect more accurately and exhaustively the co-regulated genes. More precisely, to confirm that there exists a relationship between two gene expressions, we cross two hypothesis tests. The first one is based on a nonlinear correlation computation based on the Spearman’s rank correlation coefficient. We associate to this number a hypothesis test on the dependence of the considered gene expressions. When the *p*-value of this test is less or equal to a fixed threshold (0.05 or 0.01 for our study), we conclude on the possible link between these genes that must be confirmed by a second computation based on the mutual information value associated to a significance analysis.

We consider statistical significance testing for the mutual information measurement M (X, Y), where X and Y represent the random variables associated to the considered two gene expressions. The null hypothesis H0 of this test is that X and Y are independent. The Mutual Information is a measure of the variables’ mutual dependence. Here we use it to measure this dependence for every pair of genes. In this context, we consider two random variables X and Y associated to the expression of two genes among the target genes.

The expression of M (X, Y) is given by:

M (X, Y) = H (X) + H (Y) − H (X, Y), where H (X) and H (Y) are the marginal entropies and *H* (*X*, *Y*) is the joint entropy (or the Shannon entropy) of *X* and *Y*.

Here, the computation of marginal entropy is given by, for the samples (*x*_*i*_)*i* = 1,.., *n*

$$ H(X)=-{\displaystyle \sum_i^n}P\left({x}_i\right)lo{g}_2\left(P\left({x}_i\right)\right) $$

and the joint entropy is computed by

$$ H\left(X,Y\right)=-{\displaystyle \sum_i^n}P\left({x}_i,{y}_j\right)lo{g}_2\left(P\left({x}_i,{y}_j\right)\right) $$

Intuitively, mutual information measures how much knowing one of these variables reduces the uncertainty about the other. For example, if *X* and *Y* are independent, then knowing *X* does not give any information about *Y* and *vice versa*. So their mutual information is zero. At the other extreme, if *X* is a deterministic function of *Y* and *Y* is a deterministic function of *X,* then all information conveyed by *X* is shared with *Y*: knowing *X* determines the value of *Y* and *vice versa*. As a result, in this case the mutual information is the same as the uncertainty contained in *Y* (or *X*) alone, *i.e.* the entropy of *Y* (or *X*).

First we estimate the distribution of the mutual information under H0. The main problem using the mutual information measurement is that we do not have a “reference” to say that from a certain value (0.8 for example) the two variables are dependent. In order to decide whether or not the two variables are dependent, we have to make a hypothesis test using the experimental data compared to randomly generated data. These surrogate series of data are obtained by permuting the elements of one of the studied gene expression. Thus, we compare the obtained Mutual Information results: if the one obtained by using the original computation is significantly high w.r.t. the generated ones, we conclude to the dependence of the two variables (here: gene expressions).

**Fig. 3:**
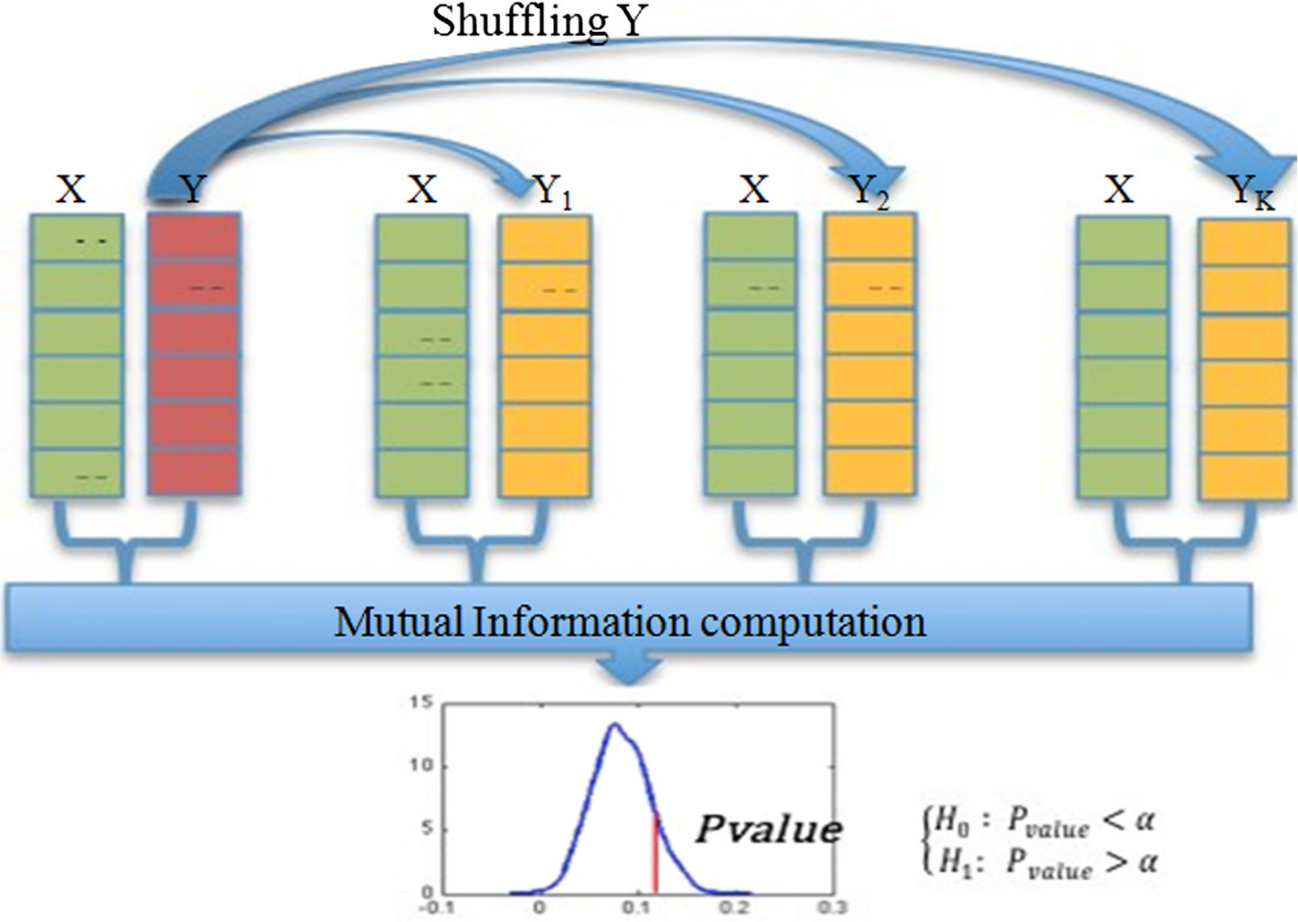
Significance analysis method. Considering the mutual information value for two data vectors, we used a shuffling method on one of these two vectors to estimate the distribution of the mutual information as a random variable. The significance test consists in comparing the obtained value of mutual information for the considered non shuffled data vectors to a function of the standard deviation. Dependence test between two random variables X and Y associated to two gene expressions: By shuffling the data of gene Y (random row permutations), we compared the obtained Mutual Information M (X, Y) results. If the one obtained by using the original computation was significantly high (*p*-value < a, which is in our case equal to 0.01) w.r.t. the generated ones, we concluded to the dependence of the two variables. Thus we could conclude on the independence hypothesis of the two data vectors. X (green), Y (red): Original data. Y_1_, Y_2_, Y_K_: Surrogate data (yellow)

Importantly, these surrogates are computed from the same number of observations, and the same distributions for X and Y (Fig. 3). We can then determine a one-sided *p*-value of the likelihood of our observation of the mutual information *i.e.* the probability of observing a greater mutual information value than that actually measured assuming H0. This can be done either by directly counting the proportion of surrogates or assuming a normal distribution of the mutual information and computing the *p*-value under a z-test.

For a given *p*-value, which is often 0.05 or 0.01, indicating that the observed results would be highly unlikely under the null hypothesis H0, we reject the latter hypothesis concluding then that a significant relationship between the two gene expressions does exist.

From these networks, we evaluate the pertinence for a unique gene to be assimilated to a breast tumor classifier in three steps. First, after choosing the gene and a classification threshold to separate the samples into two categories, we identify two networks connecting the gene to separate markers by using nonlinear correlation and mutual information techniques. Then, we define and compute the distance between the two networks, which takes into account both the structural differences between the networks (existence or not of relations between the markers, sense of the linking when it exists) and the compartmental differences (behavioral differences in the relationship between genes). Therefore, the distance between both networks represents the classification performance of the classifier gene and allows us finding the more pertinent classifiers.

## Additional files

Additional file 1: Table S1A.
*P*-values given for each gene pair in the [ER+] tumor gene network. Additional file 2: Table S1B.
*P*-values given for each gene pair in the [ER-] tumor gene network. Additional file 3: Table S2A.
*P*-values given for each gene pair in the [ERα36+] tumor gene network. Additional file 4: Table S2B. Correlation values given for each gene pair in the [ERα36+] tumor gene network. Additional file 5: Table S3A.
*P*-values given for each gene pair in the [ERα36++] tumor gene network. Additional file 6: Table S3B. Correlation values given for each gene pair in the [ERα36++] tumor gene network. Additional file 7: Table S4. Primer list.

## Abbreviations

CXCR4: Chemokine (C-X-C motif) receptor 4
DDB2: Damage-specific DNA binding protein 2
EGFR: Epidermal growth factor receptor
ER: Estrogen receptor alpha
ERα36: Estrogen receptor alpha 36
[ERα36++]: High estrogen receptor alpha 36 expression
[ERα36+]: Low estrogen receptor alpha 36 expression
ERα66: Estrogen receptor alpha 66
[ER+]: Estrogen receptor alpha 66 positive status
[ER-]: Estrogen receptor alpha 66 negative status
ERK: Extracellular signal-regulated kinases
ESR1: Estrogen receptor 1
GPER: G protein-coupled estrogen receptor 1
HER2: Human epidermal growth factor receptor 2
MAPK: Mitogen-activated protein kinases
MMP9: Matrix metallopeptidase 9
PI3K: Phosphatidylinositol-4, 5-bisphosphate 3-kinase
PR: Progesterone receptor
RANKL: Receptor activator of nuclear factor kappa-B ligand
RPLPO: Large ribosomal protein
SNAIL1: Snail family zinc finger 1
VIM: Vimentin
