# Supplementary figures and images for: From ERα66 to ERα36: a generic method for validating a prognosis marker of breast tumor progression
Source: BMC Syst Biol. 2015 Jun 17;9:28. doi: 10.1186/s12918-015-0178-7 (PMC4469423; doi:10.1186/s12918-015-0178-7)

**Additional table 1A: P-values given for each gene pair in the [ER+] tumor gene network.**

**
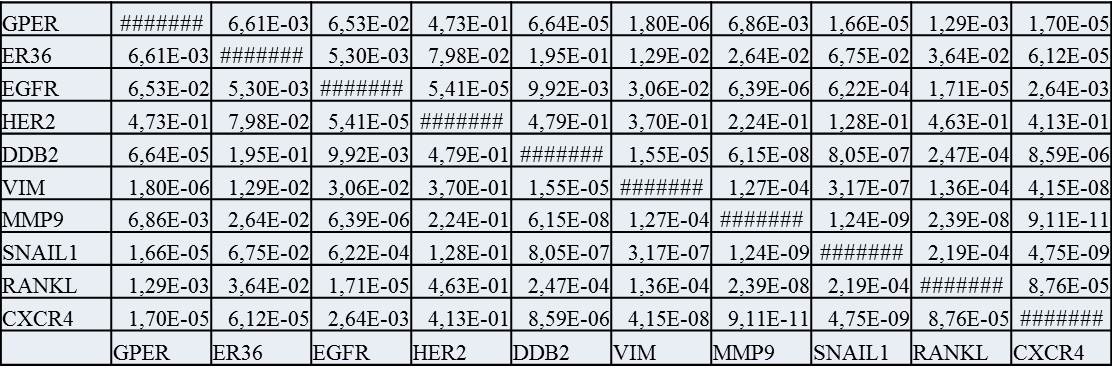
**

Supplement: Additional file 1: Table S1A. — P-values given for each gene pair in the [ER+] tumor gene network. [file 12918_2015_178_MOESM1_ESM.docx]

**Additional table 1B : P-values given for each gene pair in the [ER-] tumor gene network.**


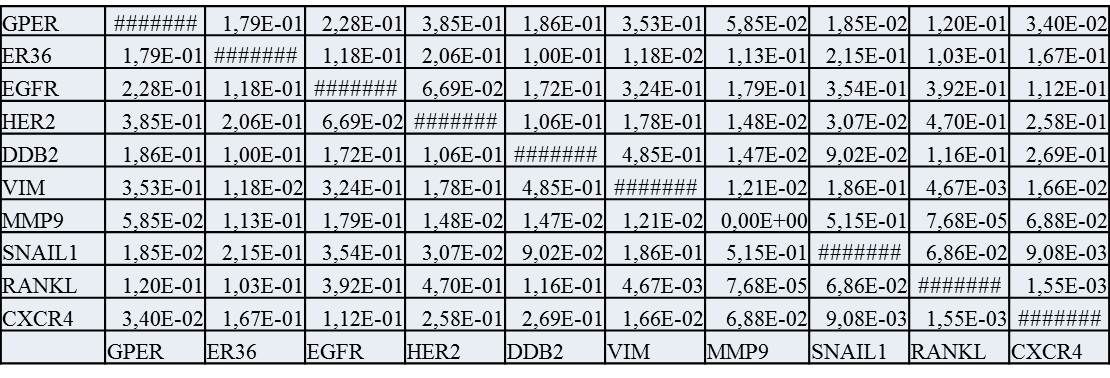

Supplement: Additional file 2: Table S1B. — P-values given for each gene pair in the [ER-] tumor gene network. [file 12918_2015_178_MOESM2_ESM.docx]

**Additional table 2A: P-values given for each gene pair in the [ERα36±] tumor gene network.**


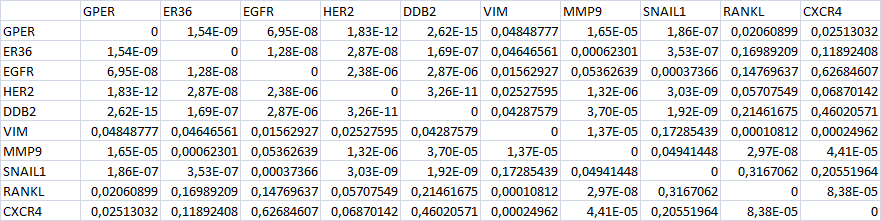

Supplement: Additional file 3: Table S2A. — P-values given for each gene pair in the [ERα36+] tumor gene network. [file 12918_2015_178_MOESM3_ESM.docx]

**Additional table 2B: Correlation values given for each gene pair in the [ERα36±] tumor gene network.**


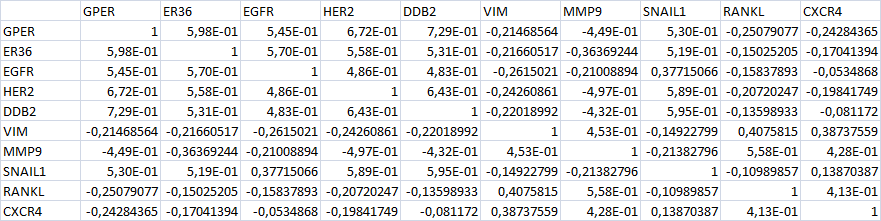

Supplement: Additional file 4: Table S2B. — Correlation values given for each gene pair in the [ERα36+] tumor gene network. [file 12918_2015_178_MOESM4_ESM.docx]

**Additional table 3A: P-values given for each gene pair in the [ERα36++] tumor gene network.**


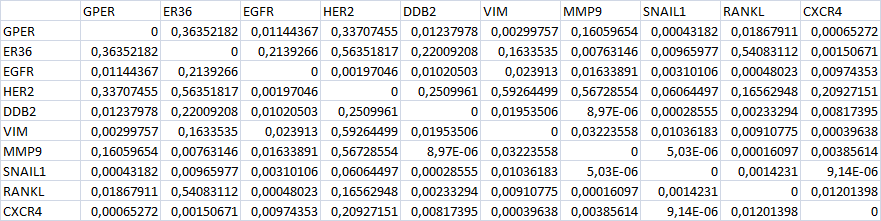

Supplement: Additional file 5: Table S3A. — P-values given for each gene pair in the [ERα36++] tumor gene network. [file 12918_2015_178_MOESM5_ESM.docx]

**Additional table 3B: Correlation values given for each gene pair in the [ERα36++] tumor gene network.**


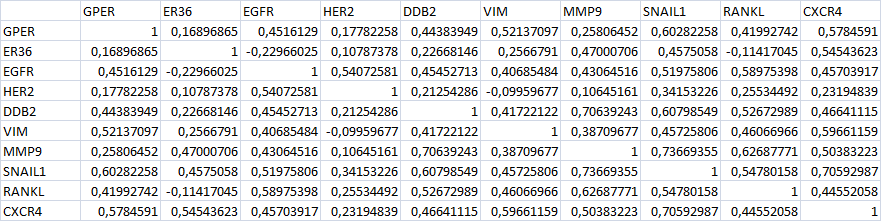

Supplement: Additional file 6: Table S3B. — Correlation values given for each gene pair in the [ERα36++] tumor gene network. [file 12918_2015_178_MOESM6_ESM.docx]
