# Supplementary material for: From ERα66 to ERα36: a generic method for validating a prognosis marker of breast tumor progression
Source: BMC Syst Biol. 2015 Jun 17;9:28. doi: 10.1186/s12918-015-0178-7 (PMC4469423; doi:10.1186/s12918-015-0178-7)
Supplement: Additional file 7: Table S4. — Primer list. [file 12918_2015_178_MOESM7_ESM.docx]

**Additional table 4 : Primer list.**

| **Gene name** |  | **Primer sequence (5’->3’)** | **Tm** |
| --- | --- | --- | --- |
| **RPLPO** | Direct  Reverse | GGCGACCGTGAAGTCCAACT  CCA TCA GCA CCA CAG CCT TC | 57°C |
| **ERα36** | Direct  Reverse | CAA GTG GTT TCC TCG TGT CTA AAG  TGT TGA GTG TTG GTT GCC AGG | 57°C |
| **GPER1** | Direct  Reverse | CTG GGG AGT TTC CTG CTG A  GCT TGG GAA GTC ACA CCA T | 57°C |
| **EGFR** | Direct  Reverse | CGT CCG CAA GTG TAA GAA  AGC AAA AAC CCT GTG ATT | 57°C |
| **ERBB2** | Direct  Reverse | AGG GAG TAT GTG AAT GCC  GGC CAC TGG AAT TTT CAC | 57°C |
| **SNAI1** | Direct  Reverse | TCT AGG CCC TGG CTG CTA CAA  ACA TCT GAG TGG GTC TGG AGG TG | 60°C |
| **RANKL** | Direct  Reverse | TCG TTG GAT CAC AGC ACA TCA  TAT GGG AAC CAG ATG GGA TGT C | 60°C |
| **CXCR4** | Direct  Reverse | ACT GAG AAG CAT GAC GGA CAA G  GAA GGG AAG CGT GAT GAC AAA G | 60°C |
| **DDB2** | Direct  Reverse | ACC AGT TTT ACG CCT CCT CAA TGG  TTT CCC TCT AAC CTG GCG CAG GTC | 60°C |
| **MMP9** | Direct  Reverse | GAG AAC TTT GCC GTT GAA GC  TCC AGC AGC TTC CTG TAG GT | 62°C |
| **VIM** | Direct  Reverse | GGG ACG CAG ACA TCG TCA TC  TCG TCA TCG TCG AAA TGG GC | 67°C |
